# Affinity of Oxybenzone and Avobenzone toward Lipids in Model Membrane Systems: On the Role of Membranes in the Harmful Effect of UV Filters on Living Organisms

**DOI:** 10.1021/acs.langmuir.6c02433

**Published:** 2026-06-02

**Authors:** Karolina Olechowska, Beata Wyżga, Katarzyna Hąc-Wydro

**Affiliations:** Jagiellonian University, Faculty of Chemistry, Gronostajowa 2, Kraków 30-387, Poland

## Abstract

Avobenzone (Avo) and oxybenzone (Oxy) are organic UV filters that are consistently released into the environment and extensively studied in terms of their toxicity and bioaccumulation potential. This study aimed to verify the affinity of these compounds and their mixture to model membranes (lipid monolayers) and to discuss the potential role of lipids in the harmful effects of Avo and Oxy on living organisms. The experiments were performed for structurally different lipids building the membranes of, e.g., mammals, fish, or crustaceans: phosphatidylcholine, phosphatidylethanolamine, sphingomyelin, and cholesterol, and for a monolayer imitating adipocyte membranes. On the basis of the surface pressure/area isotherms, the penetration studies, and Brewster angle microscopy, the following conclusions were drawn. Avobenzone and oxybenzone affect the monolayer properties by increasing their fluidity, decreasing stability, and inducing morphological changes. Although Avo reveals a significantly stronger effect than Oxy, both filters show selectivity in their interactions with the lipids. The studies on the mixed monolayers indicate that not only the lipid type but also their content in the membrane correlates with the impact of UV filters. Finally, the direct influence of Avo and Oxy on the biomembrane may not be the ground for their toxicity mechanism; however, their affinity to particular lipids may correlate with their bioaccumulation potential.

## Graphical abstract

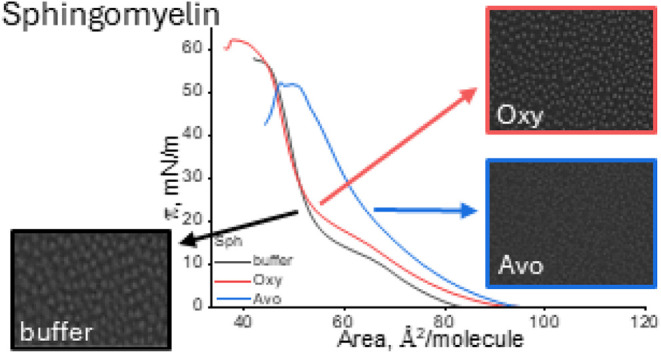

## Introduction

The sun is certainly the source of life for Earth, and exposure to sunlight has a direct impact on humans’ health. The most important benefits of spending time in the sun are the synthesis of vitamin D in the skin and increased serotonin production.^1,2^ On the other hand, it is necessary to emphasize that overexposure to the ultraviolet radiation (UVR) emitted by the sun has harmful effects on human health. UVR can be subdivided into UVA (315–400 nm), UVB (280–315 nm), and UVC (100–280 nm) radiation.^3^ UVC is completely filtered by the atmosphere, while UVA and UVB reach the Earth’s surface and are both associated with serious health consequences. Excessive exposure to UV radiation causes sunburn and is the main cause of premature skin aging. However, the most serious sunlight-related health risk is the possibility of skin cancer development (mainly keratinocyte carcinomas and melanomas).^4^ According to estimates, more than 75% of melanoma cases worldwide can be attributed to UV radiation.^5^ Thus, various prevention efforts are recommended to reduce skin exposure to UV radiation. One of the highly important strategies of photoprotection is the application of sunscreen products to exposed skin areas.

The active ingredients in sunscreen formulations are UV filters. They can be classified as inorganic (physical) and organic (chemical) based on their mechanism of action. Physical filters provide protection mainly by reflecting or scattering UVR, whereas chemical filters prevent skin damage by absorbing UV radiation with electron excitation to a higher energy state and then relaxation through heat release (of a negligible amount), emission of radiation of lower energy, or conformational change.^6–8^ Most UV filters do not provide protection against the entire range of UV wavelengths. Each filter has a specific UV wavelength range in which it shows the greatest radiation-blocking effect. Therefore, UV filters are identified as UVA (further divided into UVA1 or UVA2) and/or UVB filters.^4^ Thus, to ensure broad-spectrum UV protection (UVA and UVB), sunscreen products often contain a combination of several filters.

Among the substances currently approved for use in sunscreens are avobenzone (Avo) and oxybenzone (Oxy). The structures of these compounds are shown in Figure 1.

**Figure 1:**
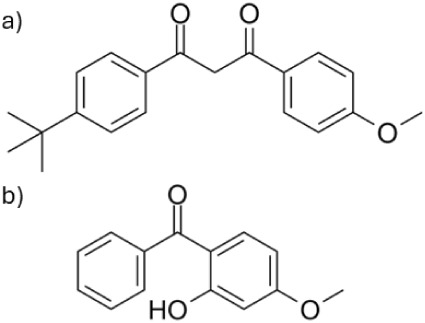
Structures of avobenzone (a) and oxybenzone (b).

Avobenzone (1-(4-*tert*-butylphenyl)-3-(4-methoxyphenyl)­propane-1,3-dione) is a dibenzoylmethane derivative considered to be a broad-spectrum UV filter. It provides protection in the UVA2 (320–340 nm) range and, as one of two chemical filters (alongside Ecamsule) approved in the European Union and United States, in the UVA1 (340–400 nm) range.^4^ Avobenzone, in the ground state, exists in two tautomeric forms: the enol form (which absorbs in the UVA wavelength range and is responsible for the sunlight protection effect) and the diketo form, with the distribution between the two forms depending on the solvent used. In sunscreen formulations, the enol tautomer is favored. On exposure to light, Avo undergoes keto–enol tautomerism and loses its photoprotective character (the diketo form absorbs in the UVC wavelength range).^9,10^

Oxybenzone (2-hydroxy-4-methoxyphenyl)­(phenyl)­methanone, benzophenone-3, BP-3), the compound belonging to benzophenones, provides broad-spectrum UV coverage in the UVA2 and UVB range^4^ and is one of the most frequently used ultraviolet filters in cosmetic products.^11^ Oxy is considered relatively stable under UV light, even after prolonged exposure to UV light.^12^

Despite these substances having been used in cosmetic products for decades, there are doubts about their safety for humans. Several studies indicate that chemical UV filters present in sunscreens, when applied to the skin, are absorbed into the stratum corneum, penetrate through the skin, and eventually enter the bloodstream. Human toxicokinetic studies show that after systematic topical application, avobenzone and oxybenzone are present in plasma and urine samples, and concentrations of these substances in plasma are reported to be above the safety norms established by the U.S. Food and Drug Administration.^13–15^ Studies on tissue samples obtained intraoperatively reveal that Oxy is not excreted fully via urine but partially remains within the organism and accumulates in adipose tissue.^16,17^ Recently, oxybenzone has also been detected in prostatic tissue.^18^ Another important issue related to the use of Avo and Oxy in sun protectors is their potential endocrine-disrupting effect.^19–22^

Exposure of living organisms to UV filters is not only caused by the direct application of cosmetics containing these substances. Namely, UV filters are emitted into the environment and are identified in various environmental elements, especially in aquatic systems. They are introduced either directly by releasing them from the skin (e.g., during swimming) or during industrial and municipal discharges (see, e.g., refs 23,24 ). The issues concerning the effects of different UV filters, including those studied herein, Oxy and Avo, on the environment—their persistence, toxicity, and bioaccumulation potential—are systematically investigated and reported in the literature. The available information allows us to consider that these hazardous chemicals pose a real threat to living organisms (e.g., refs 23–29 ).

The concentrations of these compounds and their metabolites in environmental samples vary and depend on several factors, including the type of filter, the environmental conditions, and the geographical region. In addition, the level of these contaminants fluctuates seasonally (being higher in summer) and varies from sample to sample. As indicated by the literature data, the concentrations of particular UV filters in environmental samples may reach values from ng/L to mg/L. For example, the concentration of organic filters in seawater from Gran Canaria was ca. 170 μg/L,^24^ and ca. 500 μg/L in French Mediterranean beach water,^30^ while for coral reef content, the concentration of oxybenzone in the U.S. Virgin Islands ranges from 75 μg/L to even 1.4 mg/L.^31^

Furthermore, it should be noted that in environmental samples, these contaminants usually occur together; thus, in fact, the total UV filter concentration is equal to the sum of particular substances. From a physicochemical point of view, the organic UV filters are highly hydrophobic compounds characterized by relatively high values of the octanol/water partition coefficient—*K*_*ow*_ (log *K*_*O* *W*_ = 4.5 for Avo and log *K*_*O* *W*_ = 3.6 for Oxy).^32,33^ This allows us to assume a strong affinity of these substances to lipids and fats. This fact should not be neglected when discussing the basis of the toxic effects of these substances on living organisms and their bioaccumulation potential. The latter was the ground of the research undertaken in this study. Namely, taking into account that the lipid membrane is the first barrier in the way of xenobiotics entering the cell, it can be assumed that this structure may play a significant role in the interactions of Avo and Oxy with cells. Furthermore, the membrane may determine the incorporation of UV filters into the cell and their further activities at the cellular level. Thus, for the multifaceted evaluation of the influence of the above-described UV filters on living organisms, their potential membrane effects should also be taken into consideration.

The aim of our investigation was to verify the affinity of Avo and Oxy to structurally different membrane lipids and the lipid mixture of a defined composition. Avobenzone and oxybenzone were selected as representative organic UV filters due to their widespread use and environmental occurrence, as well as their distinct physicochemical properties (lipophilicity and molecular structure), which enable a comparative assessment of their interactions with membrane lipids. The experiments focused on phosphatidylcholine (POPC), phosphatidylethanolamine (POPE), sphingomyelin (Sph), and cholesterol (Chol). These lipid classes are important structural elements building the membranes of various organisms, including mammals, fish, and crustaceans.^34–39^ A useful approach to perform the investigations, considering the plasma membrane as an important cellular structure associated with the cytotoxic effects of Avo and Oxy, is the application of model systems. Lipid monolayers at the air–solution interface are widely employed as model membranes, reproducing selected physicochemical features of biomembranes while enabling precise control of experimental conditions. Monitoring changes in monolayer compressibility and phase behavior allows us to assess, e.g., intermolecular interactions, lipid ordering, and domain formation. Such parameters are related to membrane fluidity and structural integrity, which are determinants of biomembrane permeability and cellular homeostasis. Therefore, analyzing how Avo and Oxy induce fluidization or disruption of lipid films provides mechanistic insight into membrane-level processes.

In this study, the Langmuir monolayer technique was used to systematically evaluate the influence of avobenzone and oxybenzone on the properties of the above-mentioned lipid films and to indicate the lipid component that may play a crucial role in the process of the possible incorporation and interaction of the studied chemicals with membranes. The investigations were performed for one-component lipid monolayers and, additionally, for a quaternary system. The mixed lipid monolayer was applied to reflect the multicomponent nature of natural membranes. However, its composition mimicked the adipocyte cell membrane, since, as mentioned earlier, adipose tissue is supposed to be the area of accumulation of Oxy in organisms. The experiments were carried out for Avo and Oxy used separately, as well as for the Avo/Oxy mixture, as these two UV filters are often found together in sun protection products and are present together in environmental samples. Since combined toxic effects were suggested for mixtures of organic UV filters,^40,41^ this work also addresses this problem. According to the available literature, the problem raised in this work has never been studied with the application of model membranes. Thus, the obtained results may contribute to a better understanding of the role of membranes as cellular structures, which can be involved in the harmful effects of the organic filters exerted on cells, their toxicity, and accumulation.

## Experimental Section

### Materials

The following lipids, 1-palmitoyl-2-oleoyl-*sn*-glycero-3-phosphocholine (POPC), 1-palmitoyl-2-oleoyl-*sn*-glycero-3-phosphoethanolamine (POPE), and egg yolk sphingomyelin (Sph), were purchased from Avanti Polar Lipids. Cholesterol (Chol) and the UV filters used in the studies: 1-(4-methoxyphenyl)-3-(4-*tert*-butylphenyl)-1,3-propanedione (avobenzone, Avo) and 2-hydroxy-4-methoxybenzophenone (oxybenzone, Oxy) were obtained from Merck. All the compounds were products of high purity (≥99%). The stock solutions of POPC, POPE, and Sph were prepared by dissolving appropriate amounts of these substances in a mixture of chloroform and methanol (9/1 v/v). The cholesterol solution was prepared in chloroform, whereas Avo and Oxy stock solutions were made in ethanol. The concentrations of the lipid solutions ranged from 0.3 to 0.4 mg/mL, whereas those for Avo and Oxy were 0.01 mol/dm^3^. The applied high-purity organic solvents (99.5%, HPLC grade): chloroform, methanol, and ethanol were purchased from Merck. The salts used for phosphate-buffered saline (PBS) preparation: sodium chloride, potassium chloride, sodium dihydrogen phosphate, and disodium hydrogen phosphate, were of a purity >99% and were purchased from Avantor Performance Materials. To prepare PBS and in all experiments, ultrapure Milli-Q water with a resistivity of 18.2 MΩ·cm was used.

### Surface Tension Measurements

The surface tension (γ) of Avo and Oxy solutions was measured by the Du Noüy ring method using a Krüss tensiometer (model K9). The UV filter stock solutions, at a concentration of 0.01 mol/dm^3^, were prepared in ethanol, then diluted in PBS to the desired concentration. Before each measurement, the ring was cleaned with ethanol, ultrapure water, and heated to red color with a burner. The measurements were performed at a constant temperature (20 ± 0.1 °C) and were repeated at least three times to obtain consistent results. The measurement accuracy was ±0.1 mN/m. The surface tension was measured for pure PBS buffer and for Avo and Oxy solutions at concentrations in the range of 0–50 and 0–100 μmol/dm^3^, respectively (for Avo concentrations above 50 μmol/dm^3^, aggregation was observed).

### The Lipid Systems Studied

In the experiments, POPC, POPE, Sph, and Chol monolayers, as well as their mixture, were investigated. The composition of the mixed film was estimated based on literature data regarding the content of particular lipids and fatty acid chains in mammalian adipocytes^42^ and it was as follows: 60 mol % of POPC, 25 mol % of POPE, 10 mol % of Sph, and 5 mol % of Chol. All the monolayers were spread on buffer (as the control probe) and on solutions containing Avo, Oxy, or their mixture. In the studies on the model adipocyte membrane, the concentrations of Avo and Oxy used separately were 1, 2.5, and 5 μmol/dm^3^ (that is ca. 310, 776, and 1551 μg/dm^3^ for avobenzone and 228, 570, and 1141 μg/dm^3^ for oxybenzone). The equimolar Avo/Oxy mixture (the concentration of the mixture was 5 μmol/dm^3^) was chosen based on the content of these substances in sunscreens available on the market. Namely, the concentrations of these substances in sun protection products are usually 3% (by mass) for Avo and from 2% to 6% (by mass) for Oxy. It should also be kept in mind that living organisms are exposed to mixtures of UV phytochemicals, which, as such, are found in the aquatic environment.

### Langmuir Monolayer Experiments

The experiments were performed on the KSV-NIMA Langmuir–Blodgett trough (total area of 275 cm^2^) with two Delrin barriers enabling symmetrical compression of the monolayer. As the subphase, PBS buffer or PBS buffer containing UV filters (Avo, Oxy, and Avo/Oxy mixture of molar ratio 1/1) was used. The ionic strength of the buffer was 15.4 mmol/dm^3^, and pH = 7.4. The subphase containing Avo, Oxy, or the Avo/Oxy mixture was prepared by adding appropriate volumes of filters stock solutions to PBS buffer (the volume of ethanolic solutions introduced into 250 mL of PBS was 124 μL at most, and this amount of ethanol had no influence on the course of the isotherms). The experiments were carried out for one-component POPC, POPE, Sph, and Chol monolayers and a ternary POPC/POPE/Sph/Chol system containing 60, 25, 10, and 5 mol %, respectively. Mixed solutions of the desirable composition were prepared from the respective stock solutions of the lipids. Appropriate volumes of the chloroform solutions were deposited onto the subphase with a Hamilton microsyringe (±1.0 μL). The monolayer was left for 5 min to ensure complete evaporation of the solvent and then compressed by the movable barriers at a constant rate of 10 cm^2^/min. The changes in the surface pressure (π) during monolayer compression were automatically recorded (with an accuracy of ±0.1 mN/m) using a Wilhelmy plate made of filter paper (ashless Whatman Chr1) connected to an electrobalance. The experiments were carried out at 20 ± 0.1 °C, and the temperature was controlled by a Julabo thermostat with a circulating water system. Each experiment was repeated at least two times, and the uncertainty in the estimation of the mean molecular area (*A*) was 0.5 Å^2^/molecule.

### Penetration Studies

In these experiments, the same KSV-NIMA Langmuir–Blodgett trough was used as described in section *Surface Tension Measurements*. The trough has a rectangular dipping, originally dedicated for LB deposition, where a magnetic stir bar was placed. A magnetic stirrer, integrated with the trough by a common interface, was located directly below the bottom of the well. The monolayer was formed at the air/PBS interface, compressed up to the target surface pressure, and left to equilibrate to the initial surface pressure (π_*in*_). Then, an appropriate amount of ethanolic solution of a UV filter or a filter mixture (of a final concentration in the subphase equal to 5 μmol/dm^3^) was injected into PBS with the microsyringe, usingf a Teflon injection port. The moment of insertion of the filter solution into the subphase was considered as *t* = 0. During the experiments, the subphase was continuously stirred to facilitate the distribution of avobenzone and/or oxybenzone molecules. After the injection, the changes in the surface pressure (at constant mean molecular area) caused by the penetration of filter molecules into the monolayer were recorded as a function of time. The measurements were carried out until the equilibrium surface pressure (π_*eq*_) was reached or for maximum an hour. As evidenced in the preliminary experiments, pure ethanol (in a dose analogous to that used for Avo and Oxy injection) does not affect the surface pressure values.

To ensure the reproducibility of the results, each penetration experiment was performed at least two times. The standard deviation for the equilibrium surface pressure did not exceed 2 mN/m.

The collected data were presented as Δπ vs time plots (Δπ is the difference between the surface pressure measured after the injection of the filter solution and the initial surface pressure). The positive values of Δπ mean incorporation of injected molecules into the film; Δπ = 0 indicates the absence of detectable penetration, while Δπ < 0 indicates a net decrease in surface pressure relative to the initial value, which may result from desorption of the injected compound from the monolayer, compression-induced structural reorganization, or the possibility of a partial loss of monolayer material into the subphase.^43^

### Brewster Angle Microscopy

Brewster angle microscopy (BAM) experiments were performed using the UltraBAM instrument (Accurion) integrated with a KSV-NIMA trough (total area of 587 cm^2^), equipped with two Delrin barriers and placed on an antivibration table (Standa) appointed with an active vibration isolation system (VarioBasic 40, Accurion). The microscope was equipped with a 50 mW laser emitting p-polarized light at a wavelength of 658 nm. The spatial resolution of the microscope was 2 μm. Other experimental conditions were as described for π/*A* isotherm registration.

### Computational Estimation of Cross-Sectional Area

The molecular dimensions and cross-sectional area of avobenzone in its diketo form were estimated by using Avogadro 2 (version 1.99.0). The initial 3D structure of Avo was obtained from PubChem. To obtain the most energetically stable conformation, the initial structure was subjected to geometry optimization by using the MMFF94 force field.

## Results and Discussion

### The Surface Activity of UV Filters

In preliminary experiments, the surface activity of avobenzone and oxybenzone was examined. In Figure S1, the surface tension as a function of UV filter concentration in the solution is depicted. As can be seen, in both cases, with the increase of UV filter content in the solution, the surface tension slightly decreases. This drop in surface tension values for both Avo and Oxy is negligible, as for the highest concentration applied in further experiments (5 μmol/dm^3^), the γ value is only 0.5% lower than the value obtained for pure PBS buffer. Therefore, it can be stated that the surface activity of Avo and Oxy is very low.

### The Effect of UV Filters on One-Component Lipid Monolayers

The surface pressure–area (π/*A*) isotherms recorded for POPC, POPE, and sphingomyelin monolayers on buffer and on the solutions of oxybenzone, avobenzone, and their mixture (each solution at a concentration of 5 μmol/dm^3^) are shown in Figure 2, together with the compressional modulus (*C*_*s*_^‑1^) vs the surface pressure plots for these systems. The compressional modulus (*C*_*s*_^‑1^) values were calculated according to the equation:^44^

1 $$C_{S}^{-1}=-A{\left(\frac{d\pi }{dA}\right)}_{p,T}$$

where *A* is the mean area per molecule at the given surface pressure (π).

**Figure 2:**
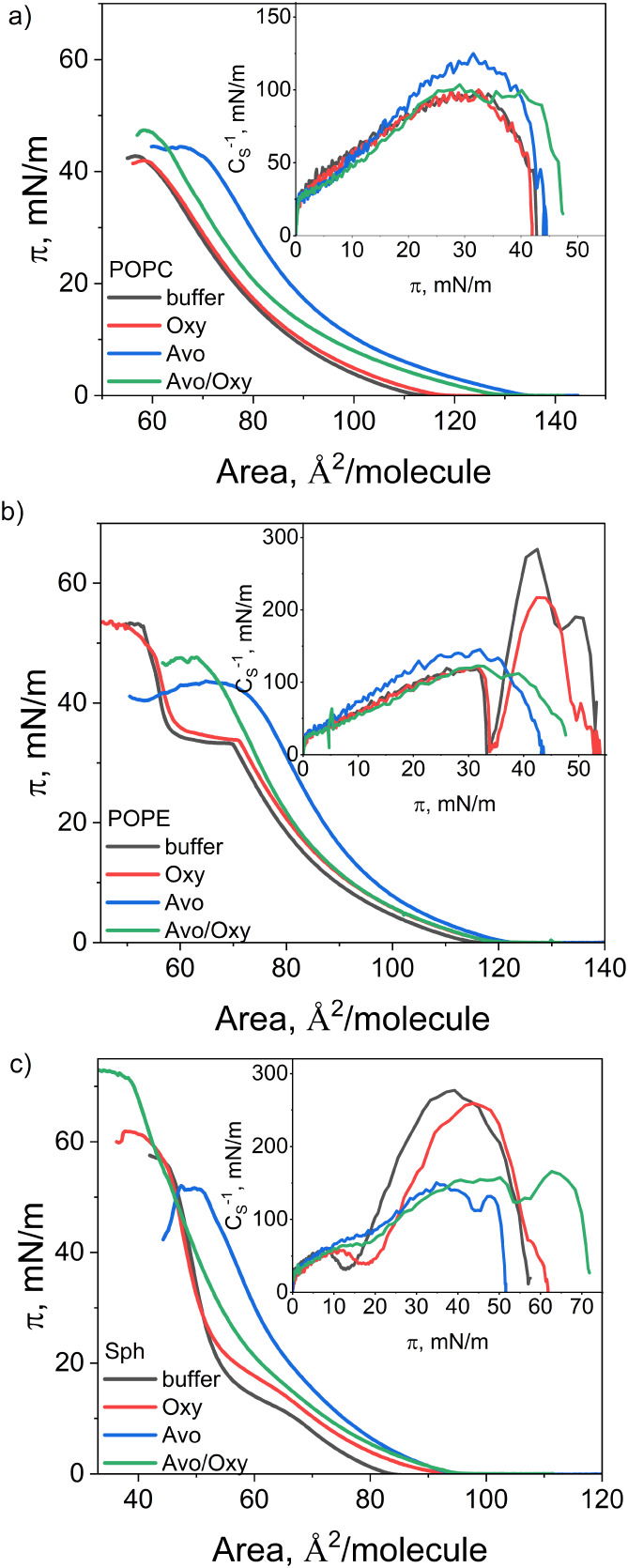
Isotherms and compressional modulus vs surface pressure plots for one-component POPC (a), POPE (b), and Sph (c) monolayers formed on buffer and on UV filter solutions at a concentration of 5 μmol/dm^3^.

The analysis of the position and course of these curves, and the values of the compressional modulus, provide information about the properties of monolayers. In short, the lower the area-per-molecule value (at a given surface pressure) and the higher the compressional modulus values, the more condensed and ordered the film.

The π/*A* and *C*_*s*_^‑1^ */π* plots obtained for POPC, POPE, and Sph on buffer stay in agreement with the data reported previously (e.g., refs. 45,46 ). Briefly, POPC forms the monolayer with the lowest condensation among the studied films, which is manifested by the largest area per molecule values, a mild π/*A* isotherm course, and low *C*_*s*_^‑1^ values (ca. 100 mN/m). In the π/*A* curve registered on the buffer subphase for the POPE monolayer, a plateau region is clearly visible (and a minimum on the *C*_*s*_^‑1^/π plot) at the surface pressure of ca. 35 mN/m, indicating a phase transition between states of varying condensation. For sphingomyelin, a bend in the isotherm, indicating a liquid expanded to liquid-condensed (LE-LC) phase transition, is observed at the surface pressure π ≈ 12 mN/m. The presence of oxybenzone in the subphase does not cause any significant changes in the course of POPC and POPE isotherms, only slightly shifting the curves to larger mean molecular area values. However, the effect of Oxy on the sphingomyelin film is more pronounced. Namely, the isotherm is visibly shifted to larger areas; however, this effect is observed only up to π ca. 25 mN/m. At higher surface pressures, the isotherms on the buffer and the curve on the Oxy solution overlap. Moreover, in the presence of oxybenzone in the subphase, the phase transition is shifted to higher surface pressures. As can be seen, in the BAM images (Figure 3), oxybenzone visibly changes the morphology of the Sph film. Namely, for the monolayer on the buffer at ca. 10 mN/m, domains of the condensed phase are formed. During compression, they grow and merge together, but the film remains inhomogeneous even above 40 mN/m. In the presence of Oxy, the condensed phase starts to form at higher surface pressures (ca. 15 mN/m), and the domains are visibly smaller than those on the buffer. These results indicate a fluidizing effect of oxybenzone on the sphingomyelin film.

**Figure 3:**
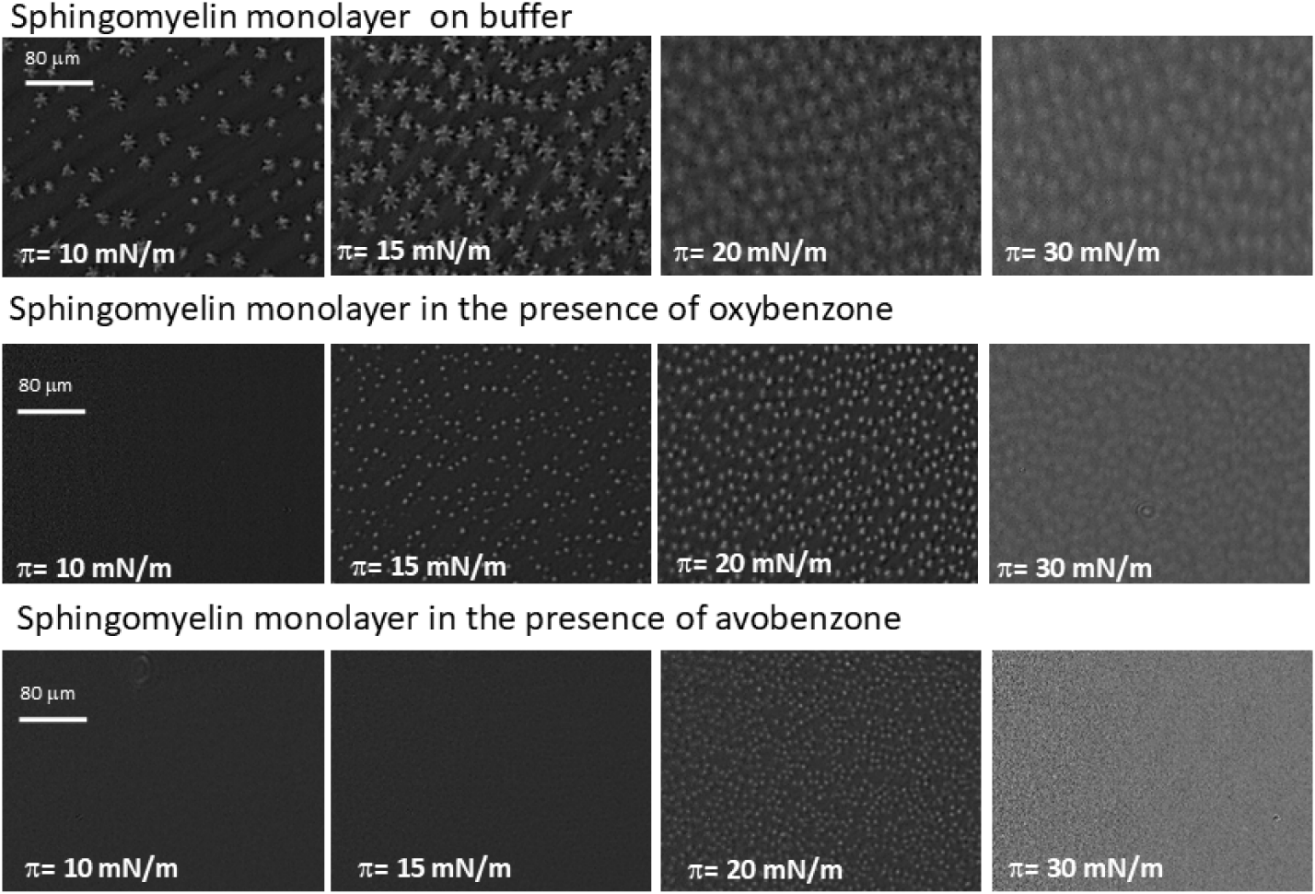
Selected BAM images for the sphingomyelin monolayer on buffer and on the solutions of oxybenzone and avobenzone.

As it can be seen, the effect of avobenzone is even stronger than the effect of oxybenzone, and it is easily observed for all of the studied phospholipid monolayers. The isotherms are shifted to larger areas. Moreover, the phase transitions observed for POPE and Sph films on buffer are not observed on Avo solution. In the presence of avobenzone, the collapse surface pressure for these two monolayers is also lower as compared to the films on buffer. All of the foregoing findings indicate that Avo causes strong fluidization as well as destabilization of these films. The fluidizing effect of this compound is also manifested in BAM images (Figure 3). Comparing the images for Sph on buffer and on avobenzone solution, it is evident that the condensed domains, which are easily observed for the film on buffer, are very difficult to identify in the presence of avobenzone, and they have significantly different morphologies.

The effect of the mixture of UV filters on the position of the curve and the collapse surface pressure is stronger than for oxybenzone and weaker than for avobenzone. For the comparison in Supporting Information (Figure S2), the shift of the curves, in percent, caused by the studied filters and their mixture, is shown. However, it should be noticed that the effect of the filter mixture on the position of the isotherms is not precisely intermediate between the effects of particular filters. Instead, the Δ*A* values are closer to those found for oxybenzone. On the other hand, when the course of the isotherms recorded in the presence of the Avo/Oxy mixture is considered, a strong impact of avobenzone can be seen. Namely, the disappearance of the phase transition for POPE and Sph films is evident, even though the concentration of Avo in the mixture is 2× lower than that used in the studies discussed above.

The last of the tested lipids was Chol. In Figure 4, the results of the surface pressure/area measurements and the compressional modulus value calculations for this lipid on buffer and on the filter solutions are presented. In the case of this film, the influence of oxybenzone is visibly stronger than that on the monolayers formed by phospholipids. The isotherm shift to the larger areas is greater, and the film is more expanded, which is also reflected in a significant drop in the compressional modulus values. Unfortunately, the impact of oxybenzone on the cholesterol monolayer was not reflected in the BAM images.

**Figure 4:**
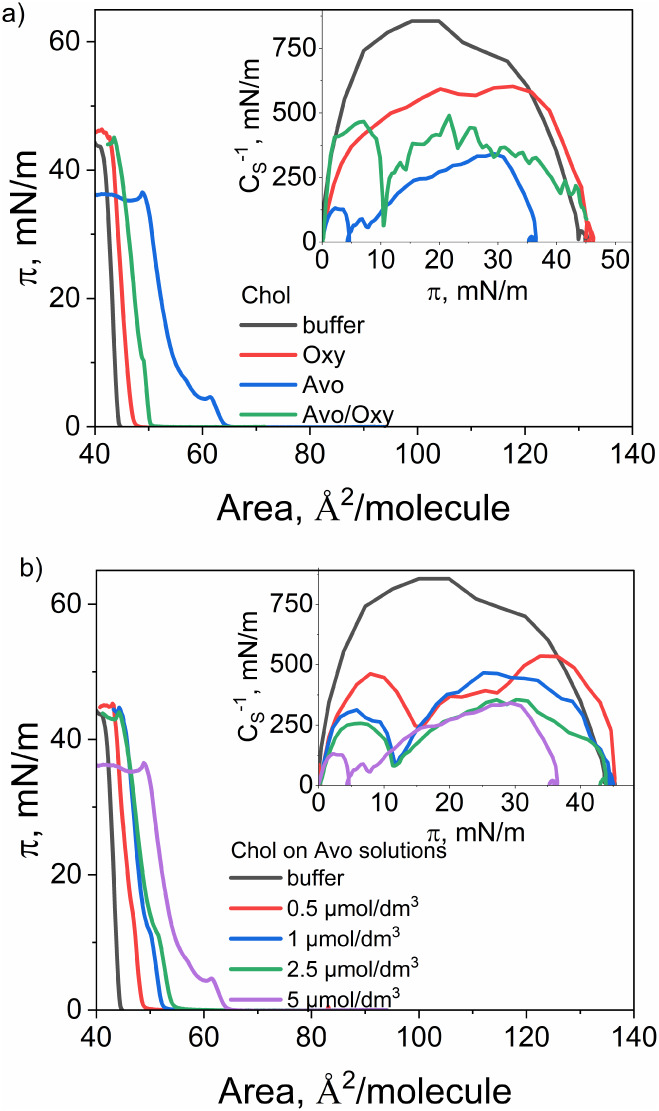
Isotherms and compressional modulus vs surface pressure plots for the Chol monolayer formed on: (a) buffer and on Oxy, Avo, and Avo/Oxy solutions at a concentration of 5 μmol/dm^3^; (b) buffer and Avo solutions of different concentrations.

Interesting results were found when the impact of avobenzone on cholesterol film was analyzed. As seen in Figure 4a, the presence of Avo in the subphase causes strong modifications in the course of the isotherm. The curve is shifted to larger areas, and the collapse surface pressure is much lower as compared to the film on buffer. The most interesting feature is the well-observable bend in the isotherm, which was not found in the phospholipid films. To get deeper insight into this effect, additional experiments for lower concentrations of Avo were performed (Figure 4b). It is evident that the bend in the isotherm appears even at a 10× lower concentration of Avo. Such an effect is not interpreted as a phase transition or the removal of some Avo molecules from the monolayer but is rather related to the strong compression-induced reorganization of the monolayer. This effect manifests as the minimum in *C*_*s*_^‑1^ vs π plot. This minimum shifts to lower surface pressure with increasing concentrations of avobenzone.

The appearance of the bend only in the π/A isotherms registered for Chol on Avo solutions can be explained by considering the unique physicochemical properties of cholesterol. While phospholipids like POPC, POPE, and Sph possess flexible acyl chains that can reorganize in the presence of Avo molecules, Chol forms rigid and highly ordered monolayers due to its planar steroid ring system. Thus, the incorporation of avobenzone molecules into such an ordered matrix at high surface pressures may promote local phase separation.

The influence of avobenzone on the cholesterol film manifests well in BAM images. This is surprising because, as mentioned above, cholesterol forms highly condensed films, and it is usually very difficult, using BAM experiments, to visualize changes in their morphology caused by the molecules in the subphase (Figure S3). The cholesterol film on the buffer is condensed and homogeneous in the whole range of compression. In contrast, in the presence of avobenzone, the BAM images reveal pronounced inhomogeneities, particularly evident at higher surface pressures. These differences indicate local variations in molecular packing and confirm the fluidizing effect of Avo on the cholesterol film. Importantly, penetration experiments demonstrate that avobenzone can insert into the cholesterol monolayer even at high surface pressures (as shown below); however, it seems that Avo molecules preferentially penetrate regions of reduced order, leading to phase separation. Thus, the differences observed in BAM images are attributed to local differences in composition and packing density induced by avobenzone insertion. It should be noted that the BAM analysis was qualitative, and no quantitative image analysis (e.g., domain size distribution) was performed in this study.

Considering the effect of the Avo/Oxy mixture on the cholesterol film, the shift of the curve and the bend characteristic of the Avo influence are well manifested. The bend (and the minimum in the *C*_*S*_^‑1^ vs π plot) is noticed at the same surface pressure as it was detected for avobenzone alone in the solution with a concentration of 2.5 μmol/dm^3^.

The affinity of UV filters for the studied lipids in their monolayers was also verified in penetration experiments. The results are shown in Figure 5. The first conclusion from these measurements is that oxybenzone has a much lower ability to incorporate into the monolayers than avobenzone. Although the surface pressure increases over time after the injection of the Oxy solution, this parameter drops below the initial surface pressure. Only for the POPC film, stabilization of the surface pressure in time was observed; however, this occurred only at lower surface pressure (10 mN/m). At 30 mN/m, oxybenzone was not inserted into any of the studied films (data not shown). Additionally, the drop of surface pressure below zero indicates the possibility of the dragging of the monolayer material into the subphase.

**Figure 5:**
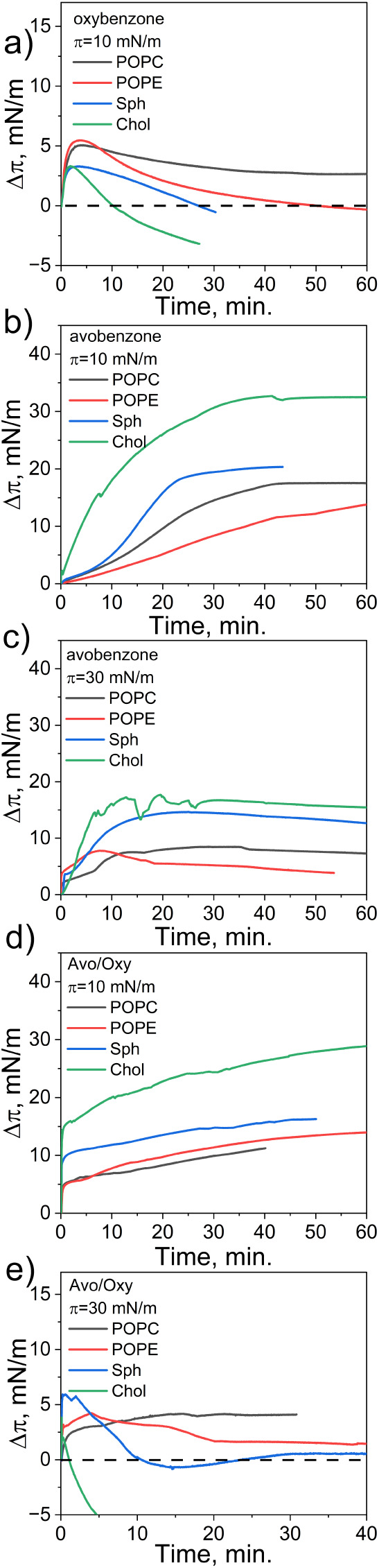
Penetration of Oxy (a), Avo (b, c), and Avo/Oxy (d, e) into one-component lipid membranes at different initial surface pressures at a concentration of 5 μmol/dm^3^.

A significantly stronger effect was observed for avobenzone. At both low and high surface pressures, Avo molecules insert into the studied films. This effect is stronger at lower surface pressure. Moreover, the penetration abilities of Avo are definitely stronger for cholesterol and sphingomyelin films, while they are weaker for POPC and POPE monolayers.

Very interesting results were obtained after the injection of the Avo/Oxy mixture. First of all, at lower initial surface pressure (10 mN/m), even after 60 min of measurement, the surface pressure did not stabilize; instead, a slow increase of this parameter was found, which means that UV filter molecules are systematically incorporated into the lipid films at these conditions. Second, analyzing these results (10 mN/m), it can be seen that the observed effect is determined strongly by the presence of avobenzone. Namely, the increase in surface pressure caused by the injection of the mixture is very high and only slightly weaker as compared to the effect of sole avobenzone, although the concentration of Avo in this mixture is 2× lower. For example, oxybenzone does not penetrate the cholesterol film (Δπ < 0), while for avobenzone, after 40 min of measurement, the surface pressure increases by 32 mN/m. For the mixture of UV filters, at the same conditions, the increase of surface pressure is ca. 26 mN/m. A similar relation was found for all the studied monolayers.

The fact that Δπ remains high despite the halved concentration of Avo suggests a near-proportional dependence of penetration capacity on the avobenzone content in the subphase, while confirming the negligible contribution of oxybenzone to the surface pressure change. This quantitative relationship highlights the high affinity of avobenzone for the rigid cholesterol monolayer, and it indicates that its interactions outweigh any potential effects of Oxy.

The third conclusion relates to the results of penetration obtained for the Avo/Oxy mixture at 30 mN/m. In this case, the penetration of UV filters was found practically only for POPC. For the remaining monolayers, the surface pressure drops below zero (cholesterol film), or it is only slightly positive. It should be pointed out that, under the same conditions, avobenzone molecules penetrate the studied films, while oxybenzone was desorbed from the monolayer. This enables us to propose that oxybenzone affects the penetration abilities of avobenzone at high surface pressure.

In Table S1 (Supporting Information), the limiting molecular area (*A*_*lim*_) values for monolayers formed on the subphase without and with Avo are presented. *A*_*lim*_ was obtained by extrapolating the steep, pseudolinear (corresponding to the condensed phase) region of the surface pressure–area isotherm to π = 0. It represents the mean molecular area of molecules at the interface under maximum packing and is used as a measure of their effective cross-sectional area in the monolayer.

### The Effect of UV Filters on the Mixed Lipid Monolayer

In Figure S4, the π/*A* isotherms for the mixed film spread on buffer and on UV filter solutions of various concentrations are shown, together with the compressional modulus vs the surface pressure plots for these systems.

The curve for the film on the buffer starts to increase at 110 Å^2^/molecules and collapses at ca. 45 mN/m. During compression, the surface pressure increases monotonically. The maximal value of the compressional modulus (130 mN/m) indicates a liquid-condensed (LC) state of the monolayer. In BAM images, at low surface pressures, the phase coexistence and the formation of the condensed phase can be noticed (Figure S5, Supporting Information). Then, up to the collapse, the condensed phase covers the interface; however, the monolayer remains not fully homogeneous.

When the monolayer is spread on solutions containing particular UV filters, the shift of the isotherms to larger areas is noticed (Figures S4 and S6). This effect is visibly the strongest for avobenzone, and additionally, in the presence of this compound, the isotherms become steeper. The latter is accompanied by an increase of the compressional modulus values. Moreover, the influence of both substances on the position of the isotherms is a linear function of their concentration (namely, the shift of the curves increases linearly with UV filter concentration in the subphase, Figure S6). Both filters did not change the morphology of this monolayer (Figure S5).

The ability of the UV filters to incorporate into lipid model membranes was verified in penetration experiments performed at various surface pressures (Figure 6).

**Figure 6:**
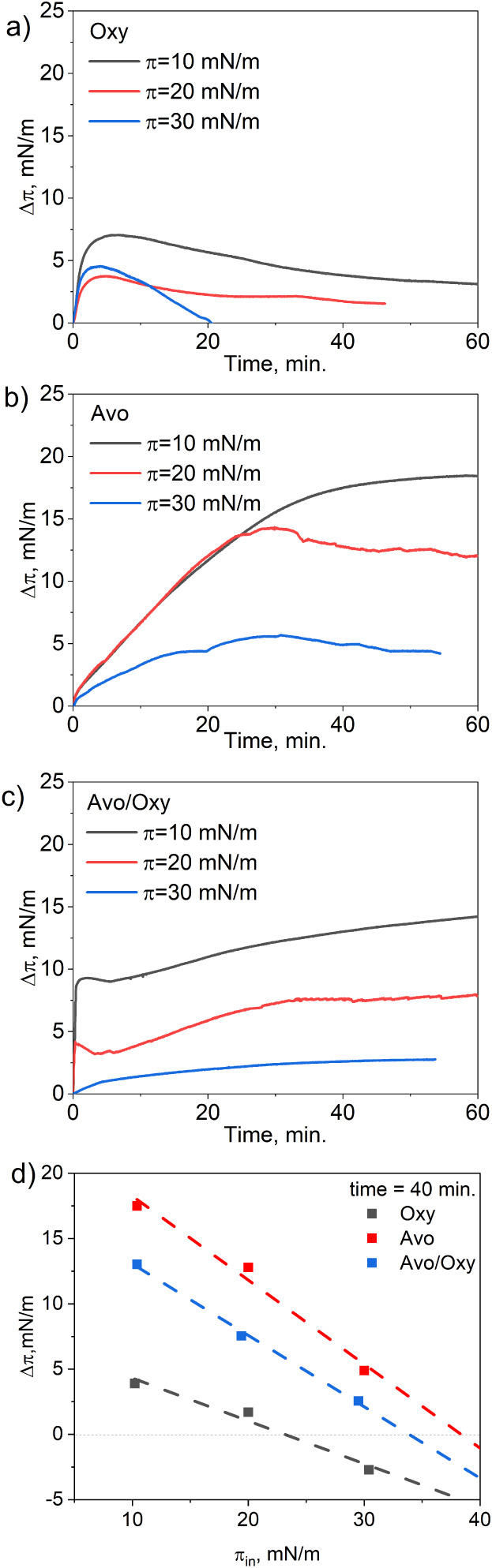
Penetration of UV filters (oxybenzone, avobenzone, and their 1:1 mixture) at a concentration of 5 μmol/dm^3^ into the mixed lipid film at different initial surface pressures (a-c); the penetration of filters vs the initial surface pressure (d).

As it is seen, Oxy is able to insert into the monolayer only at low surface pressures (namely, only at 10 and 20 mN/m, the surface pressure increased above the initial values). Avobenzone molecules incorporate into the monolayer in the whole range of the studied surface pressures. A qualitatively similar effect was found for Avo and the Avo/Oxy mixture; however, quantitatively, the influence of sole avobenzone is stronger. Figure 6d illustrates the values of Δπ at different initial surface pressures for the mixed film after the injection of particular filters and their mixture. It illustrates well that avobenzone, as well as the components of the Avo/Oxy mixture, are able to insert into this model system even in the high surface pressure region, while oxybenzone molecules incorporate into this film only up to ca. 23 mN/m.

It is interesting to compare the effects of the studied filters on the mixed monolayer and on the monolayers formed by particular lipids that are the mixture components. In Figure S7, the shifts of the isotherm (Δ*A*, %) and penetration results (Δπ, mN/m) for Oxy, Avo, and the Oxy/Avo mixture on all the studied films, obtained at the same conditions (π = 30 mN/m, concentration 5 μmol/dm^3^), are presented. When the position of the isotherms is considered, the effect of sole oxybenzone on the model adipocyte membrane (that is, the mixed film) is slightly stronger than on the remaining lipid monolayers; however, both the one-component films and the mixed system are not penetrated by this filter. For avobenzone, the effect on the position of the isotherms is very comparable for Sph, Chol, and the model adipocyte membrane. It is an important finding since Sph and Chol are minor components of the model (mixed) system (their content in the mixture is 10 mol % of Sph and 5 mol % of Chol). On the other hand, the penetration ability of this filter into the mixed system is much weaker as compared to the remaining lipids (Sph and Chol) and is similar to the penetration found for POPE and POPC. These results indicate that the composition of the membrane and the mutual proportion of particular components determine the impact of avobenzone on the system. On the other hand, the effect of the Avo/Oxy mixture on the position of the curve for the adipocyte model membrane is slightly stronger as compared to its influence on the curve for the one-component lipid films. However, the penetration after the injection of the Avo/Oxy mixture into the mixed system is rather weak.

When interpreting the results in terms of the impact of the investigated UV filters on the lipid systems, the emphasis should be placed on high surface pressures. This is due to the well-known correlation between the properties of lipids in bilayers (which form natural membranes) and in monolayers when the latter are compressed to 30–35 mN/m.^47^ We will therefore focus here on the results obtained at 30 mN/m.

The results showed differences in oxybenzone’s affinity for the lipids compared to avobenzone, which correlate well with their respective octanol/water partition coefficient (*K*_ow_) values. Specifically, avobenzone and avobenzone-containing mixtures were found to exert a significantly stronger influence (fluidization; destabilization, i.e., reduction in collapse surface pressure and inhibition of the phase transition; changes in morphology and penetration abilities) on the studied lipid systems than oxybenzone. Generally, at high surface pressures, oxybenzone molecules are not able to insert into the lipid films, which is in contrast to avobenzone molecules, which easily incorporate into all the studied monolayers. Oxybenzone causes some changes in the condensation of the films; however, this effect is significantly weaker as compared to the impact of avobenzone. This allows us to conclude that, also in the region of the lipid polar heads, avobenzone exhibits stronger activity than oxybenzone.

Although avobenzone is a hydrophobic molecule, its functional group composition enables specific interactions at the interface. As mentioned in the Introduction, the enol–keto equilibrium of avobenzone depends on the nature of the solvent.^9^ In protic media, the content of the diketo form increases due to the formation of intermolecular hydrogen bonds between avobenzone and the solvent molecules, which compete with the intramolecular hydrogen bond stabilizing the enol form, thereby promoting interactions with surrounding polar groups. This enables Avo to form hydrogen bonds with the ethanolamine group in POPE and the amide group in Sph, promoting its anchoring in the interfacial region. Because headgroup organization and hydrocarbon chain ordering are linked, such perturbations at the polar region of the monolayer propagate into the hydrophobic region, reducing the ability for chain ordering and hindering the phase transitions. In the POPC monolayer, Avo interacts with lipid headgroups predominantly through dipolar interactions (assuming that the diketo form is the predominant tautomer).

Beyond these tautomer-specific interactions of Avo, both UV filters associate with the deeper, nonpolar regions of the membrane. Although cholesterol lacks an aromatic π-system, its rigid and largely saturated sterol moiety may interact with the aromatic rings of avobenzone and oxybenzone through van der Waals and π–CH interactions. What is more, the bulky *tert*-butyl group of avobenzone is hydrophobic and can accommodate within tightly packed nonpolar regions of cholesterol and sphingomyelin films; however, its size is not proportional to the tightly packed sterol rings or hydrocarbon chains, leading to the observed perturbation of ordering and phase behavior (local phase separation and disappearance of the phase transition for Chol and Sph films, respectively).

The molecular geometry of avobenzone in its diketo form is not planar: the two aromatic rings are twisted relative to each other (dihedral angles between the ring systems are approximately −77°), and the carbonyl groups are oriented in opposite directions.^48^ Such geometry implies that the effective area occupied by the molecule strongly depends on its orientation at the interface. The calculated maximum horizontal cross-sectional area of avobenzone in its diketo form (approximately 115 Å^2^) significantly exceeds the increase in limiting molecular area values (Δ*A*_*lim*_) observed for all studied Langmuir monolayers (Table S1 in Supporting Information). This indicates that Avo does not adopt a fully horizontal orientation at the interface. Moreover, its orientation appears to be modulated by the type of the film-forming lipid. For highly ordered and condensed Chol and Sph monolayers, the relatively small increase in limiting molecular area (Δ*A*_*lim*_ = 16 and 17 Å^2^, respectively) indicates that avobenzone molecules are arranged closer to the surface normal than in less ordered monolayers. Considering the antiorientation of the carbonyl groups in Avo, simultaneous interfacial anchoring of both carbonyls is unlikely because of steric hindrance. Thus, a configuration involving one carbonyl group positioned closer to the interface is more probable. In such an arrangement, the *tert*-butylphenyl group is expected to accommodate toward the more hydrophobic, air-exposed region of the film, while the second ring, linked to the carbonyl group interfacially anchored, is positioned closer to the polar headgroup region. In contrast, a more pronounced increase in *A*_*lim*_, observed for POPC and POPE monolayers (Δ*A*_*lim*_ = 35 and 45 Å^2^, respectively), suggests that in these loosely packed environments, Avo molecules can adopt a more tilted orientation. Interestingly, the mixed monolayer exhibits a change in limiting molecular area (Δ*A*_*lim*_ = 15 Å^2^) comparable to that of cholesterol and sphingomyelin. This behavior can be attributed to the formation of sphingomyelin and cholesterol-enriched domains stabilized by specific intermolecular interactions between Sph and Chol.^49^ The high affinity between these two lipids creates a tightly packed phase—in such a raft-like environment, the avobenzone molecules may be oriented similarly as in one-component Chol and SM monolayers. While this interpretation is supported by quantitative Δ*A*_*lim*_ data, it remains indirect, as no molecular orientation measurements were performed.

In the studies on the effect of the mixture of filters on the monolayers, the following findings were collected. Both the shift of the curves, as well as the penetration abilities of the Avo/Oxy mixture, were visibly weaker than those observed for sole avobenzone; however, stronger than for sole oxybenzone. Although the changes in film condensation were not intermediated between the effects of particular filters, the strong effect of avobenzone was manifested in the course of the isotherms. On the other hand, the results of penetration experiments performed at high surface pressure evidenced the strong impact of oxybenzone on the incorporation of the filters into the monolayer. Namely, despite significant incorporation of a solely injected Avo molecule into the films under these conditions, simultaneous injection of both filters causes that none of them is inserted into the films (Chol), or the penetration is very weak. Such a strong effect of oxybenzone on the penetration abilities of avobenzone was not found at lower surface pressures.

The current research on UV filters is focused on two primary concerns: their toxicity to living organisms and their bioaccumulation potential. As regards the toxicity of the organic UV filters in the context of our experiments, two important facts should be mentioned. Namely, their negative, harmful impact on living organisms was identified even at much lower concentrations than those used in our studies.^50–54^ Second, avobenzone is considered safer for organisms than oxybenzone.^54,55^ As mentioned above, our results showed that this compound (avobenzone) has a stronger affinity for lipids than oxybenzone. Therefore, it can be postulated that the mechanism of toxicity of these two compounds may not be directly related to their harmful influence on membrane integration, and/or their interactions with membranes can be only one of the elements contributing to the toxicity of these compounds. Another important aspect of the environmental impact of UV filters is their bioaccumulation. In the experiments reported in the literature, the exposure concentrations used to study the bioaccumulation potential of these compounds were even higher than those tested in our experiments, and the results confirmed the ability of various UV filters, including Oxy and Avo, to bioaccumulate in the tissues of living organisms.^52,56^

The literature data on the tissue distribution of UV filters are scarce. However, the available results allow one to sum up that not only log *K* values but also the content of lipids may differentiate the bioaccumulation of particular UV filters in organisms. For example, the results obtained in two different studies on corals, which are rich in lipids, confirm the trend that the higher the log *K*, the stronger the bioaccumulation. However, they did not agree on the values of bioconcentration factors, which were attributed to variations in the lipid content of coral species and the time of year.^56–58^ On the other hand, in comparative studies of Oxy accumulation in different tissues (liver, gill, and muscle) and biofluids (bile and plasma) of gilt-head bream (*Sparus aurata*), the highest level of this filter was detected in bile. This finding was unexpected since, among the studied matrix, the liver is rich in lipids and should be the most favorable place for Oxy accumulation. These results were explained as a consequence of Oxy metabolization in the liver.^59^ Another study on chickens and pigeons indicates that UVA filters accumulate preferentially in the brain, kidney, and stomach over liver accumulation.^60^

The affinity of organic UV filters for particular tissues and cells, as well as the final effect induced, probably depends on the type of UV filter (i.e., the compound) and the organism.^16,56^ On the basis of our investigations, we can conclude that avobenzone readily penetrates the studied lipid monolayers. Thus, it can be proposed that the factor connected to its accumulation is the type of lipid. Results from our experiments on one-component films show that the studied UV filters exhibit selectivity toward the studied lipids: avobenzone has a stronger effect on sphingomyelin and cholesterol monolayers than on POPC and POPE films. It can be suggested that avobenzone will tend to accumulate in systems rich in cholesterol and Sph. Conversely, oxybenzone has a relatively poor effect on the organization of the studied mixed system. The penetration experiments demonstrate that Oxy is able to insert into the mixed monolayer to some extent; however, it does not remain stably incorporated in densely packed lipid films at high surface pressures, suggesting weaker stabilization within tightly ordered membrane regions. However, this is not contradictory to the confirmed accumulation ability of oxybenzone in tissues. Our observations, combined with the fact that Oxy has a low molecular weight and moderate lipophilicity (reflected in its partition coefficient), indicate passive diffusion across lipid bilayers (as described in the literature, passive diffusion is maximized for molecules that are small and moderately lipophilic^61^). Such behavior could explain the limited effect of Oxy on monolayer organization with its reported accumulation in tissues, as its ability to permeate the bilayer would allow redistribution into intracellular regions.

What is more, oxybenzone shows a stronger effect on cholesterol monolayers than on the other films at membrane-related surface pressure. This effect, occurring despite a lack of stable incorporation at high surface pressures, can be rationalized by the highly ordered and condensed character of cholesterol films. In such rigid monolayers, even temporary penetration of small amphiphilic molecules can perturb molecular packing. Thus, it seems that, in addition to passive diffusion, the lipid composition of the membrane—particularly the presence of cholesterol—modulates the effective penetration of Oxy into the interfacial region. Therefore, we propose that oxybenzone can partition into more fluid, less ordered regions of the membrane, diffuse across the bilayer, and redistribute into internal compartments.

Definitely, further research involving more types of membrane lipids found in various organisms is needed to more precisely discuss the correlation between the role of lipids in bioaccumulation and the toxicity of these filters. Moreover, since the membranes are formed by mixtures of various lipids, not only the type but also the mutual lipid proportion is important. Our results obtained for the mixed monolayer (model adipocyte membrane) seem to confirm the latter. Namely, the effect of oxybenzone on the mixed lipid film was slightly stronger than on one-component monolayers. However, for avobenzone, weaker penetration abilities and changes in morphology in the mixed film, as compared to one-component systems, were found.

Another issue related to the environmental effects of UV filters is the impact induced by their mixtures. Due to synergistic/antagonistic interactions, the effect of a mixture may differ from the effect of particular mixture components. The literature data concerning this issue are very limited. However, in the published papers, antagonistic effects between UV filters are rather postulated. For example, it was found that UV filters applied in a mixture (sunscreens) have lower toxicity toward freshwater crustaceans *Daphnia magna* than individual components applied alone.^62^ Similarly, antagonistic interactions were observed in other broad studies involving more organisms (saline crustaceans (*Artemia franciscana*), marine bacteria (*Aliivibrio fischeri*), and freshwater plants (*Lemna minor*).^63^ There are also results suggesting the additive effect of organic filters in low-dose mixtures to induce Ca^2+^ signals in human sperm cells.^40^

Our results evidenced only that the components of the 1:1 Avo/Oxy mixture at 30 mN/m exhibit very low ability to insert into the monolayer formed from particular lipids, although sole avobenzone molecules demonstrate strong incorporation potential. For the mixed monolayer, the incorporation of the components of the Avo/Oxy mixture was ca. intermediate between the effects caused by the particular filters. These results rather exclude the synergistic effects of these filters. This problem requires further investigation.

## Conclusions

The general conclusion is that the studied UV filters interact with the lipids in the monolayer; however, they are characterized by selectivity in their affinity to particular lipids. The impact of avobenzone on the lipid films is visibly stronger than the effect of oxybenzone; however, it becomes weaker in the presence of oxybenzone. Since both of these compounds are known for various harmful effects on living organisms, exerted even at very low concentrations, their toxicity mechanism may not be directly connected with the disturbances induced at the level of the membrane. However, the effect of especially avobenzone on the lipid systems can be a factor facilitating changes in the cell, leading to the confirmed toxic effects.

## Supplementary Material



## Notes

Additional experimental results and calculations (PDF)

## References

[1] Henríquez M. S., Jesús Gómez de Tejada Romero M. (2020). Cholecalciferol or Calcifediol in
the Management of Vitamin d Deficiency. Nutrients.

[2] Sansone R. A., Sansone L. A. (2013). Sunshine, Serotonin, and Skin: A Partial Explanation
for Seasonal Patterns in Psychopathology?. Innov.
Clin. Neurosci..

[3] Tobiska, W. ; Nusinov, A. ISO 21348 - Process for Determining Solar Irradiances. 36th COSPAR Scientific Assembly. Held 16 - 23 July 2006. 2006, 36.

[4] Menzie, C. A. ; Cullen, M. R. Review of Fate, Exposure, and Effects of Sunscreens in Aquatic Environments and Implications for Sunscreen Usage and Human Health; National Academies Press: Washington, DC, 2022. 10.17226/26381. 36479751

[5] Arnold M., de Vries E., Whiteman D. C., Jemal A., Bray F., Parkin D. M., Soerjomataram I. (2018). Global Burden
of Cutaneous Melanoma
Attributable to Ultraviolet Radiation in 2012. Int. J. Cancer.

[6] Manaia E. B., Kaminski R. C. K., Corrêa M. A., Chiavacci L. A. (2013). Inorganic
UV Filters. Braz. J. Pharm. Sci..

[7] Klimová Z., Hojerová J., Pažoureková S. (2013). Current Problems in
the Use of Organic UV Filters to Protect Skin from Excessive Sun Exposure. Acta Chim. Slovaca..

[8] Egambaram O. P., Kesavan Pillai S., Ray S. S. (2020). Materials Science Challenges in Skin
UV Protection: A Review. Photochem. Photobiol..

[9] Mturi G. J., Martincigh B. S. (2008). Photostability
of the Sunscreening Agent 4-Tert-Butyl-4′-Methoxydibenzoylmethane
(Avobenzone) in Solvents of Different Polarity and Proticity. J. Photochem. Photobiol., A.

[10] Vallejo J. J., Mesa M., Gallardo C. (2011). Evaluation
of the Avobenzone Photostability
in Solvents Used in Cosmetic Formulations. Vitae.

[11] Karthikraj, R. ; Kannan, K. Human Biomonitoring of Select Ingredients in Cosmetics. In Analysis Of Cosmetic Products: second Edition; Elsevier, 2018; 387. DOI: 10.1016/B978-0-444-63508-2.00015-1.

[12] Abid A. R., Marciniak B., Pędziński T., Shahid M. (2017). Photo-Stability
and Photo-Sensitizing Characterization of Selected Sunscreens’
Ingredients. J. Photochem. Photobiol., A.

[13] Lu S., Long F., Lu P., Lei B., Jiang Z., Liu G., Zhang J., Ma S., Yu Y. (2018). Benzophenone-UV Filters
in Personal Care Products and Urine of Schoolchildren from Shenzhen,
China: Exposure Assessment and Possible Source. Sci. Total Environ..

[14] Hiller J., Klotz K., Meyer S., Uter W., Hof K., Greiner A., Göen T., Drexler H. (2019). Systemic Availability
of Lipophilic Organic UV Filters through Dermal Sunscreen Exposure. Environ. Int..

[15] Matta M. K., Florian J., Zusterzeel R., Pilli N. R., Patel V., Volpe D. A., Yang Y., Oh L., Bashaw E., Zineh I., Sanabria C., Kemp S., Godfrey A., Adah S., Coelho S., Wang J., Furlong L. A., Ganley C., Michele T., Strauss D. G. (2020). Effect
of Sunscreen
Application on Plasma Concentration of Sunscreen Active Ingredients:
A Randomized Clinical Trial. JAMA.

[16] Wang L., Asimakopoulos A. G., Kannan K. (2015). Accumulation of 19 Environmental
Phenolic and Xenobiotic Heterocyclic Aromatic Compounds in Human Adipose
Tissue. Environ. Int..

[17] Artacho-Cordón F., Fernández M. F., Frederiksen H., Iribarne-Durán L. M., Jiménez-Díaz I., Vela-Soria F., Andersson A. M., Martin-Olmedo P., Peinado F. M., Olea N., Arrebola J. P. (2018). Environmental Phenols
and Parabens in Adipose Tissue
from Hospitalized Adults in Southern Spain. Environ. Int..

[18] Vitku J., Skodova T., Varausova A., Gadus L., Michnova L., Horackova L., Kolatorova L., Simkova M., Heracek J. (2023). Endocrine
Disruptors and Estrogens in Human Prostatic Tissue. Physiol. Res..

[19] Matouskova K., Jerry D. J., Vandenberg L. N. (2020). Exposure to Low Doses of Oxybenzone
during Perinatal Development Alters Mammary Gland Morphology in Male
and Female Mice. Reprod. Toxicol..

[20] Ka Y., Ji K. (2022). Waterborne Exposure
to Avobenzone and Octinoxate Induces Thyroid
Endocrine Disruption in Wild-Type and Thrαa–/–
Zebrafish Larvae. Ecotoxicology.

[21] Lee J., Kim S., Park Y. J., Moon H. B., Choi K. (2018). Thyroid Hormone-Disrupting
Potentials of Major Benzophenones in Two Cell Lines (GH3 and FRTL-5)
and Embryo-Larval Zebrafish. Environ. Sci. Technol..

[22] Huang Y., Wang P., Law J. C. F., Zhao Y., Wei Q., Zhou Y., Zhang Y., Shi H., Leung K. S. Y. (2020). Organic
UV Filter Exposure and Pubertal Development: A Prospective Follow-up
Study of Urban Chinese Adolescents. Environ.
Int..

[23] Amankwah B. K., Šauer P., Grabicová K., von der Ohe P. C., Ayıkol N. S., Kocour Kroupová H. (2024). Organic UV Filters:
Occurrence, Risks and (Anti-)­Progestogenic Activities in Samples from
the Czech Aquatic Environment and Their Bioaccumulation in Fish. J. Hazard. Mater..

[24] Cadena-Aizaga M. I., Montesdeoca-Esponda S., Sosa-Ferrera Z., Santana-Rodríguez J. J. (2022). Occurrence
and Environmental Hazard of Organic UV Filters in Seawater and Wastewater
from Gran Canaria Island (Canary Islands, Spain). Environ. Pollut..

[25] Kroll A., Kienle C., Junghans M. (2025). Organic UV-Filters
and Freshwater
Organisms: Data Gaps Impede a Robust Retrospective Environmental Risk
Assessment. Environ. Sci. Eur..

[26] Duis K., Junker T., Coors A. (2022). Review of
the Environmental Fate
and Effects of Two UV Filter Substances Used in Cosmetic Products. Sci. Total Environ..

[27] Kim S., Choi K. (2014). Occurrences,
Toxicities, and Ecological Risks of Benzophenone-3,
a Common Component of Organic Sunscreen Products: A Mini-Review. Environ. Int..

[28] Wang Y., Shang Y., Liu X., Chen X., Xu G., Lu G. (2024). Toxicity Comparison of Benzophenone-3 and Its Metabolite Benzophenone-8
in Different Tissues of Zebrafish. Aquat. Toxicol.

[29] Liu Y., Wang Y., Li N., Jiang S. (2022). Avobenzone and Nanoplastics
Affect the Development of Zebrafish Nervous System and Retinal System
and Inhibit Their Locomotor Behavior. Sci. Total
Environ..

[30] Thallinger D., Labille J., Milinkovitch T., Boudenne J. L., Loosli F., Slomberg D., Angeletti B., Lefrançois C. (2023). UV Filter
Occurrence in Beach Water of the Mediterranean Coast – A Field
Survey over 2 Years in Palavas-Les-Flots, France. Int. J. Cosmet Sci..

[31] Downs C. A., Kramarsky E. (2016). Toxicopathological Effects of the Sunscreen UV Filter,
Oxybenzone (Benzophenone-3), on Coral Planulae and Cultured Primary
Cells and Its Environmental Contamination in Hawaii and the U.S. Virgin
Islands. Arch. Environ. Contam. Toxicol..

[32] Rodil R., Moeder M. (2008). Development of a Method
for the Determination of UV
Filters in Water Samples Using Stir Bar Sorptive Extraction and Thermal
Desorption-Gas Chromatography-Mass Spectrometry. J. Chromatogr. A.

[33] Vuckovic D., Tinoco A. I., Ling L., Renicke C., Pringle J. R., Mitch W. A. (2022). Conversion of Oxybenzone Sunscreen
to Phototoxic Glucoside
Conjugates by Sea Anemones and Corals. Science.

[34] Cao X., Xu M., Feng T., Li R., Song Y., Meng N., Fan X., Zeng J., Xu J. (2024). A Comparative Lipid Profile of Four
Fish Species: From Muscle to Industrial by-Products Based on RPLC–Q-TOF-MS/MS. Food Res. Int..

[35] Zabelinskii S. A., Brovtsyna N. B., Chebotareva M. A., Gorbunova O. B., Krivchenko A. I. (1995). Comparative Investigation of Lipid and Fatty Acid Composition
of Fish Gills and Mammalian Lungs. A Model of the Membrane Lipid Component
Areas. Comp. Biochem. Physiol. B Biochem. Mol.
Biol..

[36] Chapelle S. (1977). Lipid Composition
of Tissues of Marine Crustaceans. Biochem. Syst.
Ecol..

[37] Lv S., Xie S., Liang Y., Xu L., Hu L., Li H., Mo H. (2022). Comprehensive Lipidomic
Analysis of the Lipids Extracted from Freshwater
Fish Bones and Crustacean Shells. Food Sci.
Nutr..

[38] Casares D., Escribá P. V., Rosselló C. A. (2019). Membrane Lipid Composition: Effect
on Membrane and Organelle Structure, Function and Compartmentalization
and Therapeutic Avenues. Int. J. Mol. Sci..

[39] Ali O., Szabó A. (2023). Review of
Eukaryote Cellular Membrane Lipid Composition,
with Special Attention to the Fatty Acids. Int.
J. Mol. Sci..

[40] Rehfeld A., Dissing S., Skakkebæk N. E. (2016). Chemical
UV Filters Mimic the Effect
of Progesterone on Ca2+ Signaling in Human Sperm Cells. Endocrinology.

[41] Christiansen S., Kortenkamp A., Axelstad M., Boberg J., Scholze M., Jacobsen P. R., Faust M., Lichtensteiger W., Schlumpf M., Burdorf A., Hass U. (2012). Mixtures of Endocrine
Disrupting Contaminants Modelled on Human High End Exposures: An Exploratory
Study in Rats. Int. J. Androl..

[42] Body D. (1988). The Lipid
Composition of Adipose Tissue. Prog. Lipid Res..

[43] Preetha A., Huilgol N., Banerjee R. (2006). Comparison
of Paclitaxel Penetration
in Normal and Cancerous Cervical Model Monolayer Membranes. Colloids Surf. B Biointerfaces.

[44] Davies J. T., Rideal E. K. (1961). Properties of Monolayers. Interfacial
Phenom..

[45] Węder K., Mach M., Hąc-Wydro K., Wydro P. (2018). Studies on the Interactions
of Anticancer Drug - Minerval - with Membrane Lipids in Binary and
Ternary Langmuir Monolayers. Biochim. Biophys.
Acta, Biomembr..

[46] Wyżga B., Skóra M., Olechowska K., Broniatowski M., Wydro P., Hąc-Wydro K. (2024). Searching
for the Role of Membrane
Lipids in the Mechanism of Antibacterial Effect of Hinokitiol. Arch. Biochem. Biophys..

[47] Marsh D. (1996). Lateral Pressure
in Membranes. Biochim. Biophys. Acta, Rev. Biomembr..

[48] Trossini G. H. G., Maltarollo V. G., Garcia R. D. A., Pinto C. A. S. O., Velasco M. V. R., Honorio K. M., Baby A. R. (2015). Theoretical
Study of Tautomers and Photoisomers of Avobenzone by DFT Methods. J. Mol. Model..

[49] Jiang, X.-C. Sphingolipids and Cholesterol, In Sphingolipid Metabolism and Metabolic Disease, Jiang, X.-C. , Eds.; Springer: Nature Singapore: Singapore, 2022, Vol. 1372, 10.1007/978-981-19-0394-6.

[50] Yang F., Wei Z., Long C., Long L. (2023). Toxicological Effects of Oxybenzone
on the Growth and Bacterial Composition of Symbiodiniaceae. Environ. Pollut..

[51] Miller I. B., Pawlowski S., Kellermann M. Y., Petersen-Thiery M., Moeller M., Nietzer S., Schupp P. J. (2021). Toxic Effects of
UV Filters from Sunscreens on Coral Reefs Revisited: Regulatory Aspects
for “Reef Safe” Products. Environ.
Sci. Eur..

[52] Lozano, C. ; Givens, J. ; Stien, D. ; Matallana-Surget, S. ; Lebaron, P. Bioaccumulation and Toxicological Effects of Uv-Filters on Marine Species. In Sunscreens in Coastal Ecosystems, Tovar-Sánchez, A. ; Sánchez-Quiles, D. ; Blasco, J. ; Springer, 2020; Vol. 94. 10.1007/698_2019_442.

[53] Beijora S. S., Vaz T. A. C., Santo D. E., de Almeida E. A., Junior O. V., Parolin M., da Silva Gonzalez R., de Souza D. C., Peron A. P. (2024). Prospecting Toxicity of the Avobenzone
Sunscreen in Plants. Environ. Sci. Pollut. Res..

[54] Zhong X., Downs C. A., Li Y., Zhang Z., Li Y., Liu B., Gao H., Li Q. (2020). Comparison of Toxicological Effects
of Oxybenzone, Avobenzone, Octocrylene, and Octinoxate Sunscreen Ingredients
on Cucumber Plants (Cucumis Sativus L.). Sci.
Total Environ..

[55] Santander
Ballestín S., Luesma Bartolomé M. J. (2023). Toxicity of Different
Chemical Components in Sun Cream Filters and Their Impact on Human
Health: A Review. Appl. Sci..

[56] Huang Y., Law J. C. F., Lam T. K., Leung K. S. Y. (2021). Risks of Organic
UV Filters: A Review of Environmental and Human Health Concern Studies. Sci. Total Environ..

[57] Mitchelmore C. L., He K., Gonsior M., Hain E., Heyes A., Clark C., Younger R., Schmitt-Kopplin P., Feerick A., Conway A., Blaney L. (2019). Occurrence and Distribution of UV-Filters and Other
Anthropogenic Contaminants in Coastal Surface Water, Sediment, and
Coral Tissue from Hawaii. Sci. Total Environ..

[58] Tsui M. M. P., Lam J. C. W., Ng T. Y., Ang P. O., Murphy M. B., Lam P. K. S. (2017). Occurrence, Distribution,
and Fate of Organic UV Filters
in Coral Communities. Environ. Sci. Technol..

[59] Ziarrusta H., Mijangos L., Montes R., Rodil R., Anakabe E., Izagirre U., Prieto A., Etxebarria N., Olivares M., Zuloaga O. (2018). Study of Bioconcentration
of Oxybenzone
in Gilt-Head Bream and Characterization of Its by-Products. Chemosphere.

[60] Lyu Y., He Y., Li Y., Tang Z. (2024). Tissue-Specific Distributions of
Organic Ultraviolet Absorbents in Free-Range Chickens and Domestic
Pigeons from Guangzhou, China. Environ. Res..

[61] Camenisch G., Alsenz J., Van De Waterbeemd H., Folkers G., Hoffmann F. (1998). Estimation
of Permeability by Passive Diffusion through Caco-2 Cell Monolayers
Using the Drugs’ Lipophilicity and Molecular Weight. Eur. J. Pharm. Sci..

[62] Boyd A., Martin S., Legge A., Blewett T. A. (2024). Are UV Filters Better
Together? A Comparison of the Toxicity of Individual Ultraviolet Filters
and off-the-Shelf Sunscreens to Daphnia Magna. Environ. Pollut..

[63] Stec M., Astel A. (2023). Acute Toxicity Assessment of Nine Organic UV Filters Using a Set
of Biotests. Toxicol. Res..

