# Supplementary material for: Affinity of Oxybenzone and Avobenzone toward Lipids in Model Membrane Systems: On the Role of Membranes in the Harmful Effect of UV Filters on Living Organisms
Source: Langmuir. 2026 Jun 2;42(23):17204–15. doi: 10.1021/acs.langmuir.6c02433 (PMC13362194; doi:10.1021/acs.langmuir.6c02433)
Supplement: Supplementary file 1 [file la6c02433_si_001.pdf]

The affinity of oxybenzone and avobenzone to the lipids in model membrane systems. On the role of membranes in the harmful effect of UV filters on the living organisms.

Karolina Olechowska*, Beata Wyżga, Katarzyna Hąc-Wydro

Jagiellonian University, Faculty of Chemistry, Gronostajowa 2, 30-387 Kraków, Poland

Supplementary Materials

*Corresponding author: Karolina Olechowska

karolina.weder@uj.edu.pl

0048 12 686 25 68

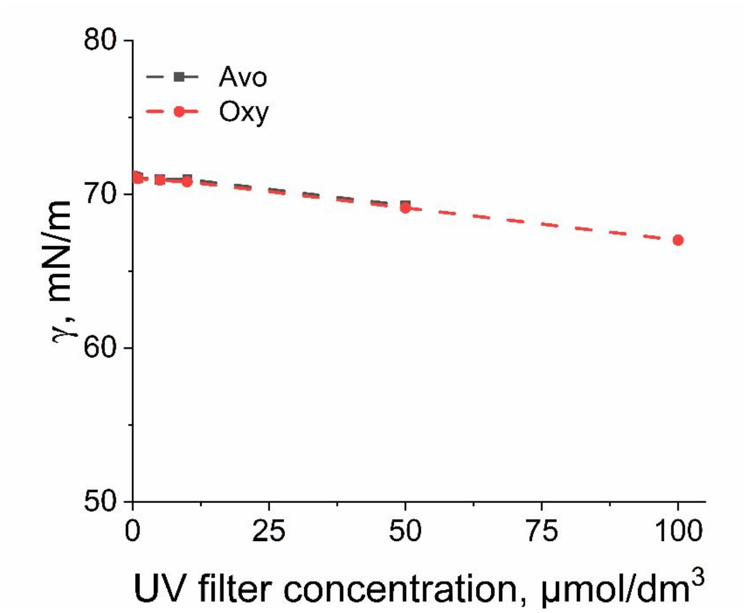

Fig. S1. The surface tension as a function of UV filter concentration.

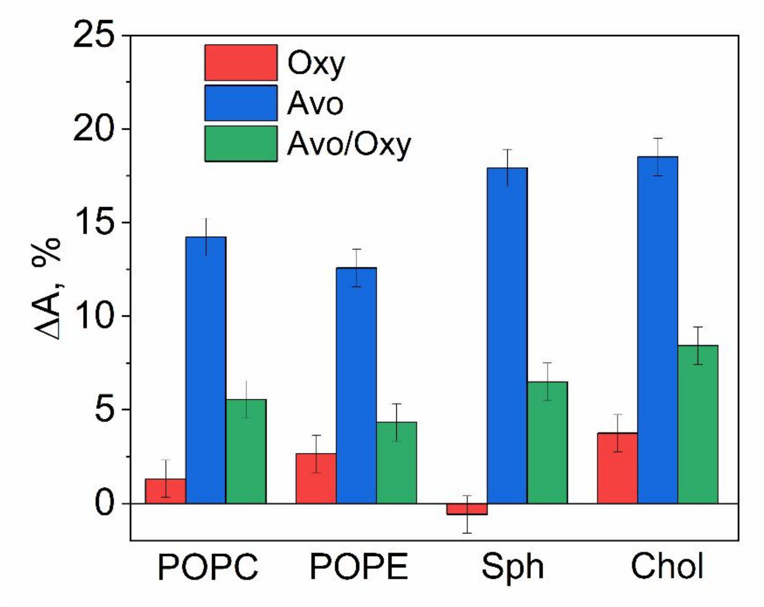

Fig. S2 The shift of the isotherms caused by oxybenzone, avobenzone and their 1:1 mixture (π = 30 mN/m)

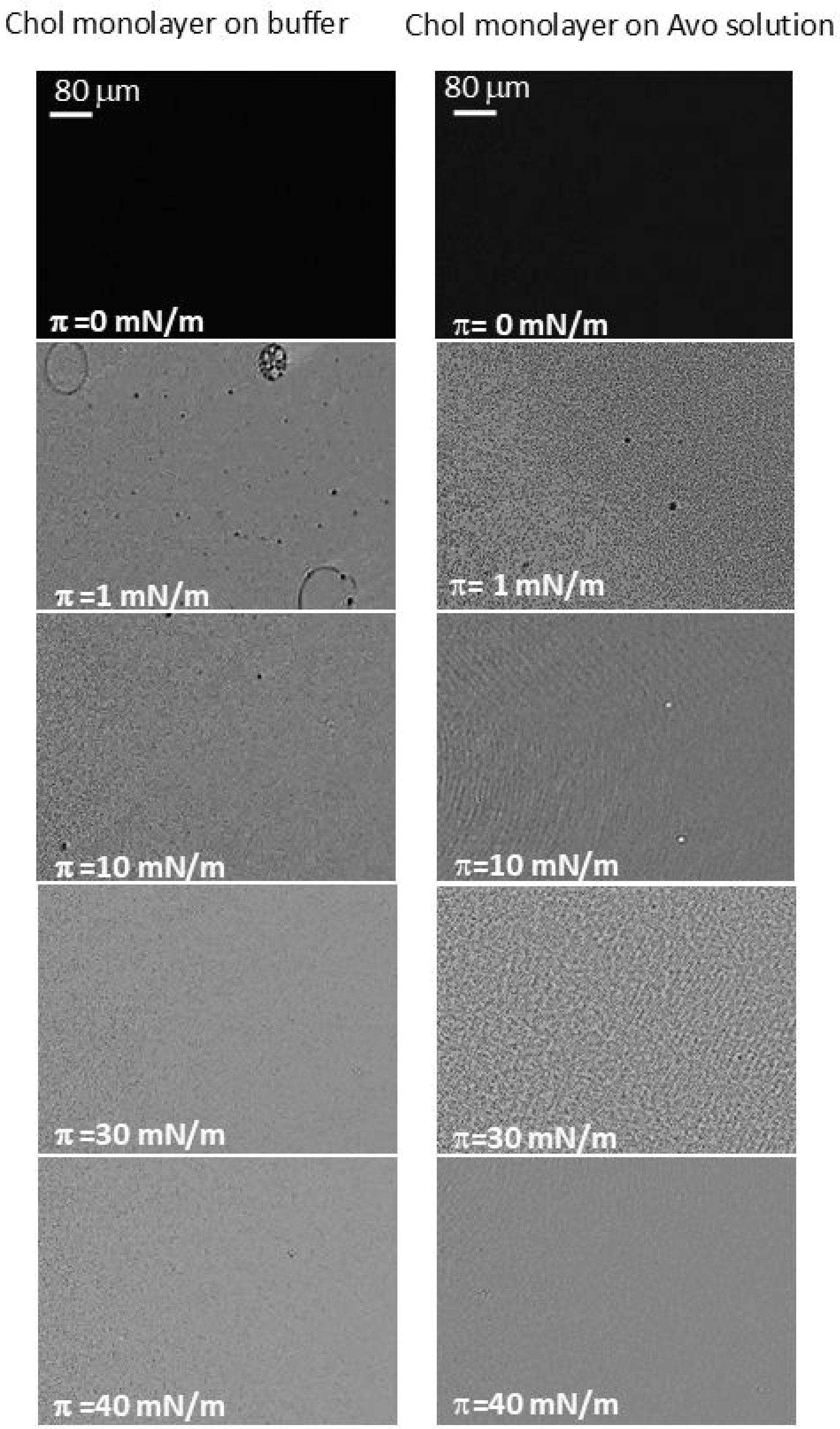

Fig. S3. BAM images recorded for cholesterol monolayer spread on PBS buffer and avobenzone solution.

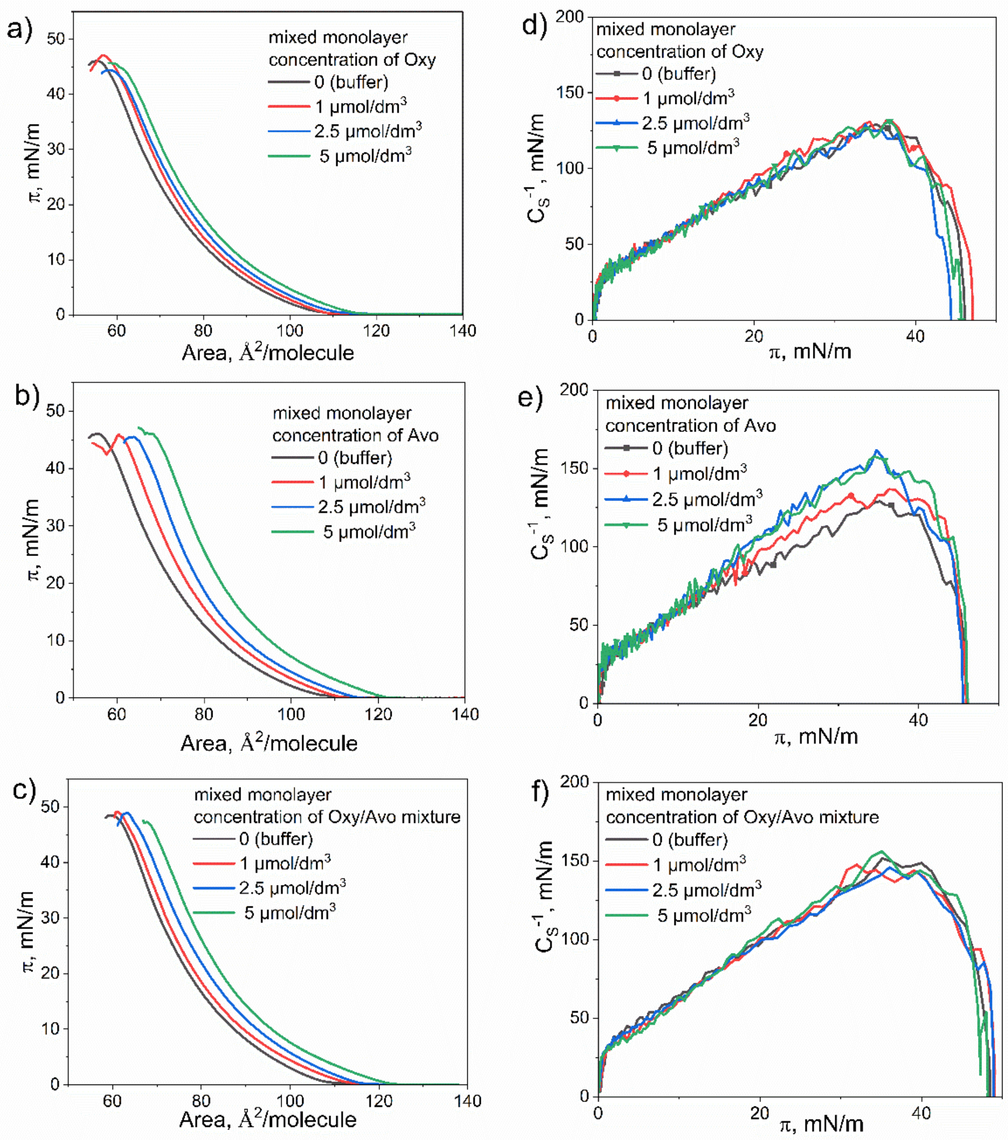

Fig. S4 The isotherms and the compressional modulus vs the surface pressure plots for the mixed lipid monolayer in the presence of oxybenzone, avobenzone and their 1:1 mixture

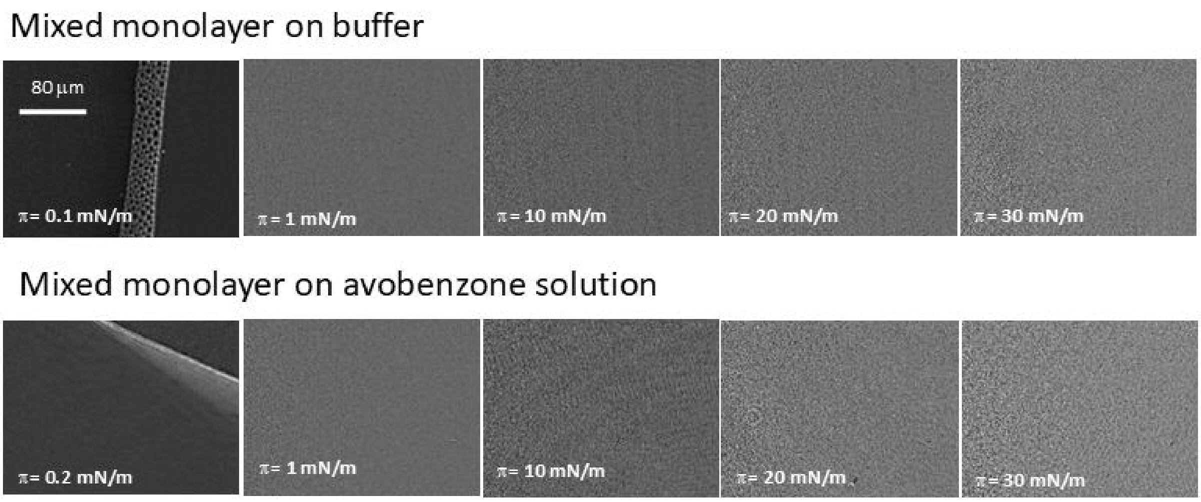

Fig. S5 BAM images for the mixed monolayer on buffer and on avobenzone solution

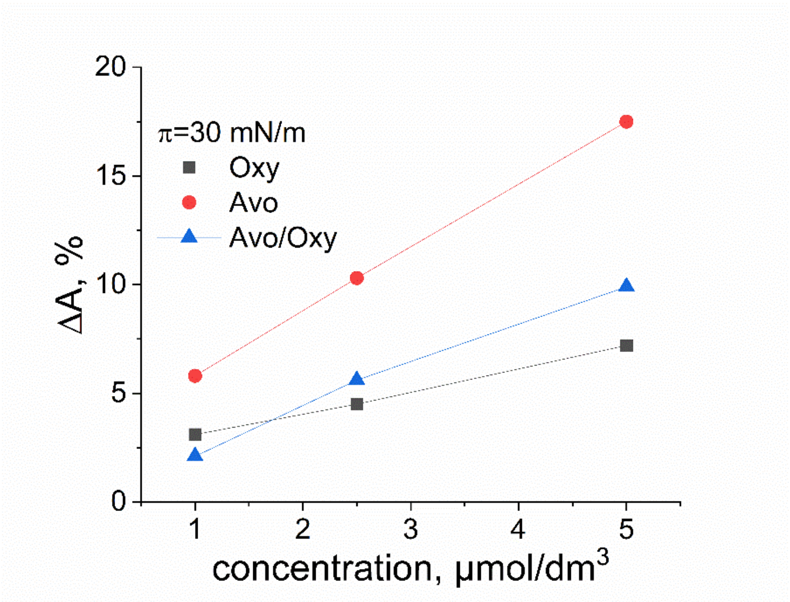

Fig. S6 The shift of the isotherm for the mixed monolayer caused by oxybenzone, avobenzone and their 1:1 mixture (π = 30 mN/m)

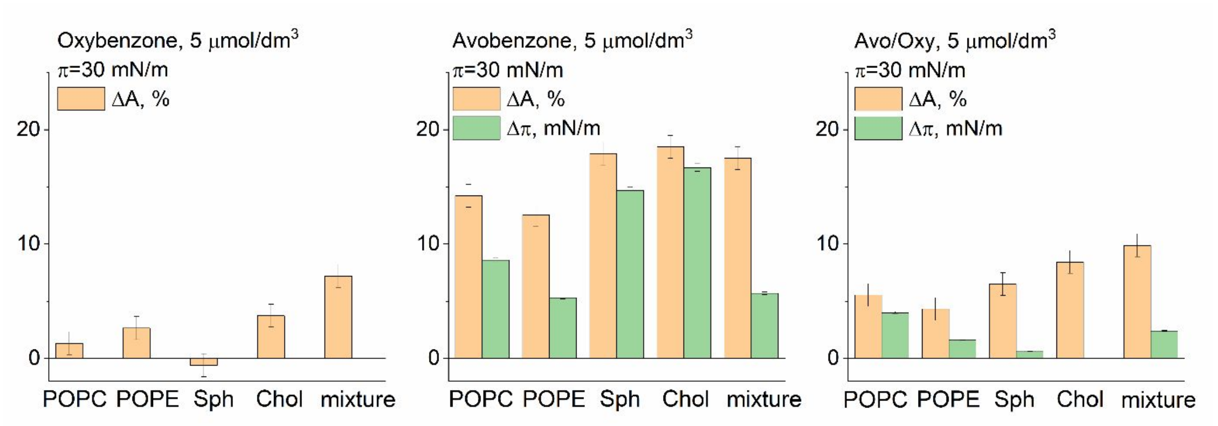

Fig. S7 The shift of the isotherm (ΔA, %) and penetration potential (Δπ, mN/m) of Oxy, Avo, and Oxy/Avo mixture on one component lipid monolayers and the mixed model adipocyte film – the comparison at the same conditions (π = 30 mN/m, concentration 5 μmol/dm^3). For Oxybenzone injected into all the systems as well as for Avo/Oxy mixture injected into cholesterol film the Δπ were negative therefore they are not presented herein

Table S1

Limiting molecular area values (A_{lim}) determined for the monolayers formed on the subphase without and with Avo (the errors of A_{lim} ± 0.5 Å^2/molecule)

	A_{lim} [Å^2/molecule]				
**subphase**	POPC	POPE	Sph	Chol	Mixed monolayer
buffer	95	65	58	44	88
5 μmol/dm^3 Avo solution	130	110	75	60	103
